# Being unvaccinated and having a contact history increased the risk of measles infection during an outbreak: a finding from measles outbreak investigation in rural district of Ethiopia

**DOI:** 10.1186/s12879-019-3973-8

**Published:** 2019-04-25

**Authors:** Abadi Girmay, Abel Fekadu Dadi

**Affiliations:** 1 Resident at Ethiopian Field Epidemiology Training Program, University of Gondar, Gondar, Ethiopia; 2 University of Gondar, College of Medicine & Health Sciences, Institute of Public Health, Gondar, Ethiopia; 3 Flinders University, College of Medicine and Nursing, School of Public Health, Adelaide, Australia

**Keywords:** Measles outbreak, Sekota Zuria District, Ethiopia

## Abstract

**Background:**

Measles is one of the most contagious diseases caused by an acute viral illness called Morbillivirus that usually occurs as an outbreak in low-income countries. As of May 2016 measles suspected outbreak was reported from Sekota Zuria district. We investigated the outbreak to identify its possible sources and risk factors of acquiring the infection in the district.

**Method:**

We conducted a 1:2 unmatched case-control study in May 2016 in Sekota Zuria district, Northern Ethiopia. Cases involved in the study were lab confirmed and epidemiologically linked. Controls were those who had no clinical signs of measles and residing in the same communities where the cases were identified. An interviewer-administered questionnaire was used to collect the data. Data were cleaned and entered to Epi-info7 and analyzed using SPSS-20. A logistic regression analysis was conducted to identify risk factors associated with measles infection at a *p*-value ≤0.05.

**Results:**

29 cases were identified during the outbreak investigation. The probable source of an outbreak was an index case who had a travel history to a district with a measles epidemic. Five samples were collected for confirmation of the diagnosis. No measles-related deaths were reported. The median age of cases and controls was 15 years (SD ± 7.8) and 11 years (SD ± 9.8), respectively. More than 55% of the cases were in age ≥ 15 years. In the multivariable analysis, being previously vaccinated for measles reduced the risk of measles infection by 83% (AOR, 95%CI = 0.17, 0.05–0.53) and having a contact history increased the risk of measles infection by 3.44 times (AOR, 95%CI = 3.44, 1.26–9.38).

**Conclusion:**

We confirmed a measles outbreak in Sekota Zuria district. The majority of the cases were in age ≥ 15 years. Being un-vaccinated and having a contact history with confirmed or suspected cases were increased the risk of measles infection. To catch up with missed children at the time of the first dose of measles vaccine and reduce their susceptibility, supplementary immunization activities (SIAs) or immunization campaigns shall be strengthened.

**Electronic supplementary material:**

The online version of this article (10.1186/s12879-019-3973-8) contains supplementary material, which is available to authorized users.

## Background

Measles is an acute viral illness caused by a single-stranded RNA virus belongs to the genus Morbillivirus [1]. Measles is one of the most contagious of all infectious diseases with > 90% attack rates among susceptible close contacts [2]. Primarily it is transmitted by respiratory droplets or airborne spray to mucous membranes in the upper respiratory tract or conjunctiva [3]. Measles cases are infectious starting from the prodromal period (when the first symptom appears) to four days after the appearance of the rash.

Measles is characterized by a generalized maculopapular rash, fever, cough, coryza (running nose), conjunctivitis, and photophobia [2]. The incubation period from exposure to the onset of fever is approximately 10–12 days and from the exposure to the onset of rash is 7–18 days [4]. Though many children experience uncomplicated measles, nearly 30% of cases may develop one or more complications that are more common in young children with immune deficiency disorders, malnutrition, vitamin “A” deficiency, and inadequate vaccination [1].

Vaccination has reduced a global measles morbidity and mortality over the last 30 years [5]. Despite these acknowledged sign of progress in morbidity reduction, measles is still not controlled in many parts of the world; particularly in Africa and Asia [6]. During the 2014 and 2015, a total of 296,629 and 206,360 measles cases reported globally [7].

In Ethiopia, the national immunization program was established in 1980 and has been delivering through static and outreach modalities [8]. The current immunization schedule recommends a routine first dose of measles vaccine at the age of nine [9]. As a sero-conversion rate of measles vaccine at 9 months of age is around 85%, a second dose opportunity through supplementary immunization activities (SIAs) or immunization campaigns [9] might be required to protect those children who have never been vaccinated or those who were vaccinated but did not develop immunity [3].

A single dose of national measles vaccination coverage was 76 and 84% in 2013 and 2014, respectively. Ethiopia has adopted the regional goal of measles mortality reduction in 2002 and measles elimination goal in 2012 [9]. Despite several efforts has been made to implement the elimination strategies, the country has been experiencing a number of measles outbreak annually. During the 2014, a total of 16,702 clinically suspected cases were notified to the Ethiopian surveillance system. Of the 13,301 suspected cases reported in 2014, 2373 (18%) were laboratory confirmed, 5692 (43%) were epidemiologically linked, and 5236 (39%) were clinically compatible [9]. Between 2000 and 2014, in the Amhara region, 2412 cases were lab confirmed, 23,842 were epidemiologically linked, and 9699 were clinically compatible [10]. As one of the 11 zones of Amhara, Wag-Himra alone reported 629 measles cases and 24 deaths [11]. As of May 2016, Sekota Zuria district health officials have notified the occurrence of suspected measles outbreak to Wag-Himra Zone public health emergency management (PHEM) department. As a response, a multidisciplinary team was deployed to the area aiming at verifying the existence of an outbreak and to identify its possible causes.

## Methods

### Study setting

Sekota-Zuria is one of the seven districts found in Wag-Himra Zone. It is located at 425 km North East to Bahir-Dar, which is the capital city of the Amhara region. The district had a total population of 135,309 in 2016. It has 33 rural Kebeles (lowest administrative level), seven health centers and 33 health posts. Zuna, which is an outbreak affected Kebele, is one of the 33 Kebeles in the district. Health centers in the district provide static immunization service while health posts serve as a tentative immunization service. Based on the Sekota Zuria district health office report (unpublished data), a one-dose measles vaccination coverage was 74.2% in 2016.

### Study design and sample size

A 1:2 unmatched case-control study was conducted from May 18 to 30, 2016 in Sekota Zuria district. A total of 87 samples**,** 29 available cases and 58 comparable controls were included from the outbreak affected Kebeles. Two neighborhood controls for each case were recruited and got enrolled in the study.

### Enrolment of cases and controls

#### Cases

Those that have clinical signs and symptoms of measles based on the case definitions in the national measles guideline that were either laboratory confirmed or epidemiologically linked to the laboratory confirmed cases [3].

#### Controls

Controls were selected from the same kebele and were neighbors of the cases but free of the disease during the outbreak investigation period.

### Data collection procedure

A house-to-house searching was done to include all available cases using a line list record of cases in the area. A standardized questionnaire was developed by the author after a thorough literature review. A set of seven questions were asked to assess study participants’ knowledge about measles and all correct responses were scored four points while each wrong response scored zero. Study participants who scored a mean and above were graded as having a “good knowledge” while those who scored below a mean were graded as having a “poor knowledge” about measles. We used an interviewer-administered questionnaire to collect socio-demographic characteristics of cases and controls, possible risk factors of the disease, immunization status, and knowledge of respondents about measles infection. Moreover, information on immunization coverage of the district and vaccine handling system (availability of refrigerator, vaccine carrier, ice pack, and cold chain management) were also collected from the district health offices and health centers Additional file 1.

### Data processing and analysis

Data were cleaned and entered to Epi-info7 and imported to SPSS 20 for further analysis. Descriptive analysis was conducted to generate percentages and figures whereas odds ratios with its 95% confidence intervals were computed to compare risk factors among cases and controls. In the multivariable logistic regression model, a *p*-value ≤0.05 and a 95%CI was computed to declare a statistically significant association between measles infection and risk factors.

### Ethical issues

The ethical approval of this study was secured from the Institutional Review Board of the University of Gondar and permission to conduct the study was obtained from Wag-Himra Zone health office. Assent or oral informed consent was obtained from all study participants and the information obtained was kept confidential.

## Results

### Epidemiological characteristics of the study participants

A total of 29 measles cases were identified during the outbreak investigation. No measles-related deaths were reported. All the five blood samples tested were positive for measles-specific-IGM antibodies while the rest 24 cases were epidemiologically linked. The median age of cases and controls was 15 years (SD ± 7.8) and 11 years (SD ± 9.8), respectively. More than 55% of the cases were 15 years and above. All measles cases were reported from one kebele (Zuna) and the majority (59%) of them were males. The overall (crude) attack rate in the kebele was 69.9/10,000 population and the highest age-specific attack rate was observed among 15–44 years (93.8/10,000).

**Fig. 1:**
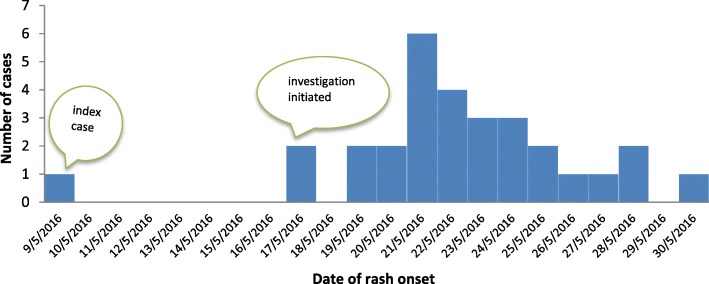
Number of measles cases by date of rash onset, Zuna kebele, Sekota Zuria district, Amhara region, May 2016

The investigation was initiated after four suspected measles cases have been reported from the district. The deployed multidisciplinary team has discussed with key informants of the kebele and affected family members about the disease condition in the community. According to the information provided, a 27-year-old woman was sick with similar signs and symptoms one week before the reported cases. The woman (suspected index case) had a travel history to Ziquala district, which is adjacent to the affected kebele, to visit her relatives before two weeks of the investigation and she has reported that her relatives were sick. As it shows in the Epi-curve, the outbreak has propagated quickly and reached a peak on May 21/2016 and then dropped gradually. No additional cases were reported after May 30/2016 despite active contact tracing has been conducted in the affected kebele (Fig. 1).

### Immunization coverage

**Fig. 2:**
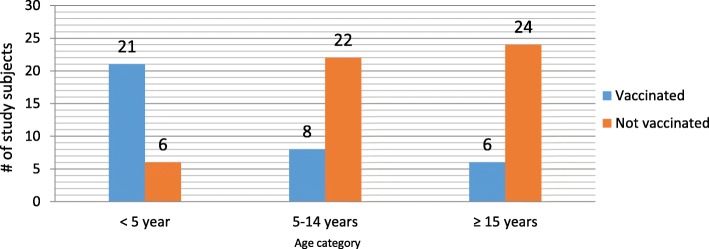
Vaccination status of study subjects by age group, Sekota Zuria district, Wag-Himra Zone, 2016

The routine one dose measles vaccination coverage of the district was 74.2% during the 2016. The proportion of vaccinated individuals was relatively low among cases than controls (17% vs. 52%). Similarly, the number of vaccinated adults were much lower than the vaccinated under-five children. (Fig. 2).

### Factors associated with measles infection

After adjusting for confounding effect, in the multivariable regression model, being previously vaccinated reduced a risk of measles infection by 83% as compared to those who were unvaccinated (Adjusted Odds Ratio (AOR), 95%CI = 0.17, 0.05–0.53). On the other hand, having a contact history with measles cases was increased the risk of measles infection by 3.44 times (AOR, 95%CI = 3.44, 1.26–9.38). (Table 1).

**Table 1:** Bi-variable and multi-variable logistic regression analysis of factors associated with measles outbreak, Zuna kebele, Sekota Zuria district, Amhara region, May 2016

Variables	Measles status		Crude odds ratio (COR)_(95% CI)_	Adjusted odds ratio (AOR)_(95% CI)_
	Case	Control		
Vaccination status				
Yes	5	30	0.19 (0.07–0.58)	0.17 (0.05–0.53)*
No	24	28	1.00	1.00
Contact history with measles cases				
Yes	18	21	2.88 (1.15–7.25)	3.44 (1.26–9.38)*
No	11	37	1.00	1.00
Educational status				
Illiterate	22	47	1.00	1.00
Primary	7	11	1.22 (0.42–3.53)	1.57 (0.38–6.56)
Occupation				
Farmer	22	51	1.00	1.00
Student	7	7	0.43 (0.14–1.38)	0.36 (0.01–1.34)
Knowledge about measles transmission				
Yes	19	40	0.86 (0.33–2.20)	0.82 (0.26–2.54)
No	10	18	1.00	1.00
No of people living in a room				
< 5	13	40	1.00	1.00
≥ 5	16	18	2.74 (1.091–6.86)	1.89 (0.65–5.56)
Age in years				
< 5	5	22	0.20 (0.06–0.67)	0.36 (0.08–1.58)
5–14	8	22	0.32 (0.11–0.94)	0.33 (0.10–1.09)
≥ 15	16	14	1.00	1.00
Sex				
Male	17	29	1.42 (0.58–3.49)	1.64 (0.52–5.21)
Female	12	29	1.00	1.00

*P<0.05

## Discussion

This study intended to assess risk factors associated with measles infection in Zuna kebele of Sekota Zuria district. The measles infection was confirmed after five serum samples were positive for measles specific-IgM antibodies. A measles outbreak was established after three or more lab- confirmed cases were found in the district within a month [3]. Other cases were epidemiologically linked as the outbreak was localized to those laboratory-confirmed cases. None of the cases had a history of traveling to other measles risky areas prior to 7–18 days disease onset but all cases have acquired the infection at least eight days (the minimum incubation period) after the index case has got sick in the kebele they were living. As measles is highly contagious, studies suggested that a single case may infect 17–20 susceptible people and a secondary attack rate near to 75–90% has been reported [4].

No measles related deaths were reported in this study though case fatality from measles is estimated to be 3–5% in developing countries and may reach more than 10% when occurred in nutritionally deprived areas [3]. The absence of death in this study might be due to an effective case management following an early detection of the outbreak and because of the fact that the majority of the cases were adults. The majority of the cases (55.2%) in this study were adults aged ≥15 years. This might be due to the accumulation of unvaccinated adults in the community as they were not targeted for immunization program unlike that of children under-five years. Other findings have also showed a shift in age distribution to adult cases despite the disease was more prevalent among children prior to expansion of immunization programs [1, 12]. The 2014 Ethiopian annual report on measles have showed that 67% of the measles cases of the year were above five years [13]. Another report on epidemiologic data also showed a decreasing proportion of measles cases in children under five and outbreaks continue to occur in most parts of the country with nearly 70% of the reported cases being 15 years and above [9].

The overall incidence rate in the study area was 69.9/10,000 and relatively higher attack rate (93.8/10,000) was observed among 15 years and above. This rate is higher than the attack rate recorded nationally during the measles outbreaks in 2013 and 2014, which was 6.5 and 14.6 per 100,000 population, respectively [13]. Even though the national measles vaccination coverage has generally increased over time (44% in 2003 to 84% in 2014), it is far from the standard coverage in which more than 90% vaccine coverage is required to reduce possible outbreaks [2]. The same is true in the case of Sekota Zuria district in which the vaccination coverage was low (74.2%) and the vaccination rate of the study participants was low (40%).

Our multivariable analysis revealed that previously vaccinated individuals had 83% less risk of acquiring measles infection as compared to unvaccinated individuals. This result is consistent with the theoretically calculated vaccine-preventable fraction which showed 85% protective vaccine efficacy when an individual receives one dose of Measles-Containing Vaccine (MCV) at 9 months of age [3]. This finding is also similar to another study which found that vaccinated individuals had less risk to acquire measles infection [14].

We also found that people who had a contact history with measles cases before 2–3 weeks prior to developing the current infection had a 3.4 times higher risk of acquiring measles infection compared to those people who had no known contact history during the same period. This finding is consistent with the study conducted in Bugna and Kindo Didaya districts of Ethiopia in which susceptible individuals who had a contact history with measles cases had a higher risk of acquiring measles infection as compared to individuals who had no known contacts [15, 16].

As a limitation, we only found 29 measles cases that seems small, however, it has been documented that case-control study is efficient with relatively small sample size in general and especially it is highly recommended in case of an epidemic as compared to other observational studies [17]. This work might also subject to recall bias as some study participants lack immunization cards and were asked to recall their immunization status.

### Prevention and control measures taken

All cases were provided with a supportive treatment with vitamin “A”, tetracycline (TCC) ointment, and oral rehydrating salts (ORS) as early as possible and severely ill patients were taken to the nearest health center for better supportive treatment and follow up. Health information was provided to the community to create awareness towards the importance of vaccination. Active case search was conducted to find out additional cases and to assess the progress of the interventions in the affected area. Moreover, a brief discussion was made with a surveillance focal persons of the district aiming to strengthen the surveillance system of the area.

## Conclusion

The result of this investigation assures the occurrence of measles outbreak in Sekota Zuria District. Vaccination coverage among cases was lower than among controls. More than half of the total cases were adults’ age ≥ 15 years. High attack rate was observed in older ages. The predominant contributing factors for the occurrence of this outbreak was being unvaccinated and having a close contact history with measles cases. Increasing immunization coverage to ≥90% and strengthening Supplementary Immunization |Activities (SIAs) to refill the routine immunization schedule might reduce the number of susceptible individuals in a population and risk of an outbreak.

## Additional file

Additional file 1: Questionnaire used in outbreak investigation. (DOCX 18 kb)

## Abbreviations

AOR: Adjusted odds ratio
COR: Crude odds ratio
MCV: Measles containing vaccine
ORS: Oral rehydrating salts
PHEM: Public health emergency management
SIA: Supplementary immunization activities
TCC: Tetracycline

## References

[1] Measles in England and Wales;Measles the notifiable disease:Green Book Chapter 21; 2013.

[2] Status report on progress towards measles and rubella elimination: October 2012.

[3] Guideline on Measles Surveillance and Outbreak Management. 3^rd^ Edition. EHNRI, Addis Ababa, Ethiopia, January; 2012.

[4] Preeta K Vaccine preventable disease surveillance manual, 6^th^ Edition. 2013.

[5] Beyene BB, Tegegne AA, Wayessa DJ, Enqueselassie F (2016). National measles surveillance data analysis, 2005 to 2009, Ethiopia. Journal of Public Health and Epidemiology.

[6] WHO/ UNICEF:The Measles & Rubella Initiative; Annual Report. 2013.

[7] Reed J. Measles (Rubella) Disease Report. 2016.

[8] Ethiopia National Expanded Programme on Immunization: comprehensive multi-year plan 2011–2015,FMoH, addis ababa, December, 2010.

[9] National EPI, Comprehensive Multi-Year Plan 2016 - 2020, FMoH,Ethiopia, Addis Ababa. 2015.

[10] Getahun M, Beyene B, Ademe A, Teshome B, Tefera M, Asha A, Afework A, HaileMariyam Y, Assefa E, Gallagher K (2016). Epidemiology of laboratory confirmed measles virus cases in Amhara regional state of Ethiopia, 2004–2014. BMC Infect Dis.

[11] Wag-Himra Zone Health Office (2016). Public health emergency management(PHEM), measels surveillance annual report (unpublished).

[12] Measles reporting and surveillance guidelines. Washington State Department of Health August 2014.

[13] Expanded Programme on Immunization Annual Report, Ethiopia. 2014.

[14] Adissu A. Measles Outbreak in Adults,Tahitay Adiyabo District, Tigray Region, Ethiopia. 2012.

[15] Ayalew A (2015). Measles epidemic investigation.

[16] Zemelak E. Investigation of measles outbreak in Kindo Didaya district, southern Ethiopia,2013.

[17] Mann CJ (2003). Observational research methods. Research design II: cohort, cross sectional, and case-control studies. Emerg Med J.

