# Supplementary material for: Being unvaccinated and having a contact history increased the risk of measles infection during an outbreak: a finding from measles outbreak investigation in rural district of Ethiopia
Source: BMC Infect Dis. 2019 Apr 25;19:345. doi: 10.1186/s12879-019-3973-8 (PMC6485078; doi:10.1186/s12879-019-3973-8)
Supplement: Supplementary file 1 — Questionnaire used in outbreak investigation. (DOCX 18 kb) [file 12879_2019_3973_MOESM1_ESM.docx]

**Appendix 1: English Version Questionnaire**

| **Part one: - Socio-demographic assessment** | | | | | | |
| --- | --- | --- | --- | --- | --- | --- |
| No | Questions | | Responses | | | Code |
| 101 | Child date of birth | | ___/____/_____ | | |  |
| 102 | Age of the child | | _______months | | |  |
| 103 | Sex of the child | | 1= male 2= female | | |  |
| 104 | Birth order of the child | | ________ | | |  |
| 105 | Family size | | _________ | | |  |
| 106 | # of children ever born | | _________ | | |  |
| 107 | # of children alive | | _________ | | |  |
| 108 | Mother‘s/care giversˈ marital status | | 1= single  2= married  3= divorced  4= widowed | | |  |
| 109 | Who is the primary caretaker of the child? | | 1=Mother  2=Father  3=Other(specify)_____ | | |  |
| 110 | Age of the mother (caregiver)? | | 1=____years, 88= don’t know | | |  |
| 111 | Educational status of mother (caregiver)? | | 1=illiterate  2=read and write  3= grade 1-8  4= grade 9-12  5=college/university | | |  |
| 112 | Mother’s (caregiver’s) occupation? | | 1= House wife  2=government employee  3=merchant  4= daily labourer  5= other ( specify)___ | | |  |
| 113 | What is the religion of the primary caretaker? | | 1=Orthodox  2=Muslim  3=Protestant  4=Catholic  5=Other __________ | | |  |
| 114 | Average monthly income (in birr)? | | __________ | | |  |
| **Part-Two: Mothers/caregivers Knowledge on immunization related Questions** | | | | | | |
| 201 | Have you ever heard about vaccination or vaccine preventable disease? | | | 1=Yes  2= No | | If NO, skip to Q 203 |
| 202 | What was your source of information? (Multiple response possible) | | | 1=Radio  2=Television  3= friends  4=from school  5=Health personnel  6=other, specify _________ | |  |
| 203 | Do you attend any media(Radio or TV) | | | 1=Yes  2= No | | If NO, skip to Q 205 |
| 204 | How many times per week? | | | _________ | |  |
| 205 | Would you mention the benefit of vaccinating a child? (Multiple response possible) | | | 1=to prevent from disease  2=for child health  3=other, specify ___________  88= don’t know | |  |
| 206 | What vaccine preventable diseases do you know? (Multiple response possible) | | | 1. Measles  2. Diphtheria  3. Polio  4. Tetanus  5. Pertussis  6.Hepatitis b  7. Homophiles influenza b 8.pneumonia  9. diarrhoea  10.Tuberculosis  88= don’t know | |  |
| 207 | Would you tell me the age at which the child begins vaccination? | | | _______________  88=I don’t know | |  |
| 208 | How many vaccination sessions are needed for a child to be fully vaccinated? | | | _______________  88=I don’t know | |  |
| 209 | At what age the child should complete vaccination? | | | _______________  88=I don’t know | |  |
| 210 | Where do you take if your child gets ill? | | | 1= Health facility  2= Traditional healers  3= Holly water  4=Remains at home  5=Other(specify) | |  |
| **Part-three: Maternal Health care utilization** | | | | | |  |
| 301 | Did the mother of the baby attended antenatal care (ANC) during her last pregnancy? | | 1= Yes  2= No | | | If no, skip to Q 303 |
| 302 | How many times did you/she attend? | | ________ | | |  |
| 303 | Did the mother take TT vaccine? | | 1= Yes  2= No | | | If no, skip to Q 305 |
| 304 | How many times did you/she take? | | ________ | | |  |
| 305 | Where did the mother deliver her last child? | | 1= Home  2= Health Post  3=Health Center  4=Hospital  5=Other (specify)______ | | |  |
| 306 | Did the mother had post natal care (PNC) check ups | | 1= yes 2=No | | | If No, skip to Q401 |
| 307 | How many times did you/she? | | ___________ | | |  |
| **Part-four: Accessibility and availability of vaccination service** | | | | | |  |
| 401 | | Is there any nearby health facility that provides vaccination service? | 1=Yes  2=No | | |  |
| 402 | | If yes, which health facility is near to you? | 1=health post  2= health centre  3=hospital  5=private clinic | | |  |
| 403 | | How does it take you to reach there? | 1. Less than 15 minutes  2. 15-30 minutes  3. 30-1hour minute  4. > 1 hour | | |  |
| **Part-five: Child Vaccination History** | | |  | | |  |
| 410 | | Did your child take any vaccination? | 1=Yes  2=No | | | If no, skip to Q414 |
| 411 | | Do you have a card where vaccinations are written down? (copy vaccination data) | 1=Yes  2=No | | |  |
|  | |  | Vaccines Taken | | | dd/mm/yyyy |
|  | |  | BCG | |  |  |
|  | |  | OPV0 | |  |  |
|  | |  | OPV1 | |  |  |
|  | |  | OPV2 | |  |  |
|  | |  | OPV3 | |  |  |
|  | |  | Pentavalent 1 | |  |  |
|  | |  | Pentavalent 2 | |  |  |
|  | |  | Pentavalent 3 | |  |  |
|  | |  | Measles | |  |  |
|  | |  | PCV1 | |  |  |
|  | |  | PCV2 | |  |  |
|  | |  | PCV3 | |  |  |
|  | |  | Rota1 | |  |  |
|  | |  | Rota2 | |  |  |
| 412 | | Has a child had any vaccinations that are not recorded on this card? | 1=Yes  2= No | | | If no, skip to Q414 |
| 413 | | What are the types of vaccines? | 1= vaccine against measles  2=vaccine given against polio  3=Routine vaccines | | |  |
| 414 | | What are the reasons for defaulting? If child is a defaulter, (Multiple response possible) | 1= Vaccination site is far-away  2= Vaccination time is inconvenient  3= Absenteeism of vaccinators  4=Lack of awareness on the importance of vaccination  5= Not knowing vaccination time and site  6= Not knowing whether to come back for second and third vaccination  7= fear of side effects  8= lack of transportation  9=Others(specify)__________ | | |  |
| **Please tell me if the child had any of the following vaccinations** | | | | | |  |
| 416 | | BCG vaccination against tuberculosis, that is, an injection in the arm or shoulder that usually causes a scar | | 1=Ye  2=No | |  |
| 417 | | Polio vaccine, that is, drops in the mouth? | | 1=Ye  2=No | |  |
| 418 | | Was the first polio vaccine given in the first two weeks after birth or later? | | 1=Ye  2=No | |  |
| 419 | | How many times was the polio vaccine given | | _______ | |  |
| 410 | | A pentavalent vaccination, that is, an injection given in the left thigh? | | 1=Ye  2=No | |  |
| 411 | | How many times Pentavalent vaccination is given? | | ________ | |  |
| 412 | | A PCV vaccination, that is, an injection given in the right thigh? | | 1=Ye  2=No | |  |
| 413 | | How many times PCV vaccination is given? | | _______ | |  |
| 414 | | Was the Rota vaccine given as a drop the same day the second round polio drop is given? | | 1=Ye  2=No | |  |
| 415 | | How many times was the Rota vaccine given | | _________ | |  |
| 416 | | A measles injection that is, a shot in the arm at the age of 9 months or older – to prevent him/her from getting measles? | | 1=Ye  2=No | |  |
